# Utility of Polymerase Chain Reaction, Enzyme-linked Immunosorbent Assay, and Gene Sequencing in Detecting Orientia tsutsugamushi Infection Among Pediatric Acute Encephalitis Syndrome Cases in Northern India

**DOI:** 10.7759/cureus.81549

**Published:** 2025-03-31

**Authors:** Hricha Mishra, Amita Jain, Chandra Kanta, Shantanu Prakash, Rajkumar Kalyan

**Affiliations:** 1 Paediatrics, King George's Medical University, Lucknow, IND; 2 Clinical Microbiology, King George's Medical University, Lucknow, IND; 3 Microbiology, King George's Medical University, Lucknow, IND

**Keywords:** acute encephalitis syndrome (aes), enzyme-linked immunosorbent assay (elisa), pediatric, phylogenetic analysis, polymerase chain reaction (pcr), scrub typhus

## Abstract

Introduction

This study aims to evaluate the diagnostic performance of polymerase chain reaction (PCR), enzyme-linked immunosorbent assay (ELISA) methods, and gene sequencing in detecting *Orientia tsutsugamushi*, the causative agent of scrub typhus, in pediatric patients with acute encephalitis syndrome (AES).

Methods

The study was conducted at a tertiary care teaching hospital in northern India. In all, 264 children presenting with AES were enrolled between August 2018 and November 2019. Cerebrospinal fluid (CSF), serum, and peripheral blood mononuclear cell (PBMC) samples were analyzed using real-time PCR and ELISA to detect the presence of scrub typhus. The 56 kDa gene sequencing was performed in 15 samples.

Results

The ELISA for scrub typhus IgM demonstrated the highest cumulative positivity rate at 26.52%. In contrast, PCR testing of CSF and PBMC samples showed lower detection rates of 4.17% and 9.47%, respectively. The overall positivity for scrub typhus was 29.5%, highlighting the significant role of this pathogen in pediatric AES cases in endemic regions. The phylogenetic analysis of scrub typhus revealed many Gilliam-like strains.

Conclusion

ELISA for scrub typhus IgM has a high positivity rate in comparison to PCR for the detection of scrub typhus in children with AES.

## Introduction

Scrub typhus, caused by the bacterium *Orientia tsutsugamushi*, is a vector-borne illness that affects various countries in Asia, including India, Southeast Asia, and Japan, and is linked to high morbidity and mortality [1]. The disease is primarily transmitted through the bite of infected chigger mites and can result in a wide array of symptoms, ranging from mild febrile illness to severe complications, such as acute encephalitis syndrome (AES) [2]. Pediatric patients are particularly susceptible to severe forms of scrub typhus, including AES, which can cause long-term neurological impairments or death if not recognized and treated promptly [3].

Early and accurate detection of scrub typhus in pediatric patients with AES is critical for timely treatment management. Two commonly used diagnostic methods for this purpose are polymerase chain reaction (PCR) and enzyme-linked immunosorbent assay (ELISA). PCR is a highly sensitive molecular technique for detecting *O. tsutsugamushi* DNA in clinical samples, whereas ELISA detects pathogen-specific antibodies [4]. The type of biological sample used, such as cerebrospinal fluid (CSF), serum, or peripheral blood mononuclear cells (PBMCs), plays a crucial role in the diagnostic accuracy of these methods [5]. Gene sequencing helps in the identification of circulating genotypes of *O. tsutsugamushi* in a particular region [4,5]. 

The objective of this study was to assess the diagnostic performance of PCR and ELISA in detecting scrub typhus infection in pediatric AES cases, using CSF, serum, and PBMC samples. By comparing the diagnostic test of each method across different sample types, this study aims to establish a reliable approach for early and accurate diagnosis of scrub typhus in children with AES. We hypothesized that ELISA has a higher positivity rate than PCR in the detection of scrub typhus in these cases. Ongoing surveillance is also crucial to assess circulating *O. tsutsugamushi* strains. This study sequenced the 56 kDa gene to identify genotypes in pediatric AES cases in Uttar Pradesh and analyze their diversity. Insights gained from this research will help in improving treatment outcomes in this vulnerable population.

## Materials and methods

Study design and population

This prospective cross-sectional study was conducted at King George’s Medical University, Lucknow, India, on pediatric patients diagnosed with AES. This study was carried out over 15 months from August 2018 to November 2019. The inclusion criteria for the study were pediatric patients aged three months to 14 years who presented with clinical features suggesting of AES according to WHO protocol [6]. Children who had epilepsy, trauma, toxin exposure, tuberculous meningitis, cerebrovascular accidents, and brain malignancy were excluded. 

Ethical clearance

Ethical approval was obtained from the institutional ethics committee (89th ECM II B-PhD/P3). Written informed consent was obtained from the parents and assent from patients (when applicable) before enrolment. 

Sample size

Based on the previous studies from the same region [4], expecting that 20-25% of AES cases will be positive for scrub typhus, and taking a margin of error 5% with a confidence interval of 95%, a sample size of 246-289 was calculated.

Sample collection

Three types of biological samples, such as CSF, serum, and peripheral blood, were collected from each patient under sterile conditions within 24 hours of admission, except in a few patients, where CSF sampling was delayed due to hemodynamic instability at presentation. 

PBMC isolation from whole blood

Histopaque 1077 (Sigma Aldrich Chemicals Pvt. Ltd., Bengaluru, India) was used to isolate PBMC. A 15 mL centrifuge tube was filled with 5 mL of the Histopaque solution. After that, 4 mL of EDTA blood and an equal volume of phosphate buffer saline (PBS) were combined and gently deposited on top of the Histopaque solution. The tubes were centrifuged at 400 × g for 30 minutes at 4°C with low acceleration and low deceleration, and the PBMC layer was meticulously aspirated and washed three times with PBS. The last pellet was resuspended in 300 µL of sterile PBS and kept at −80°C for DNA extraction [7].

PCR and ELISA for *Orientia tsutsugamushi*

DNA extraction was performed on all collected samples (CSF and PBMC) using QIAamp DNA Blood Micro Kit (Qiagen, GmbH, Hilden, Germany) for PBMC and QIAamp DNA Micro Kit for CSF, according to the manufacturer’s instructions. The presence of *O. tsutsugamushi* DNA was detected using a real-time PCR assay targeting the 47 kDa protein gene [8]. The PCR reaction was carried out in a QuantStudio™ 7 Flex Real-Time PCR System (manufactured by Thermo Fisher Scientific, Waltham, MA, USA) with the following cycling conditions: initial denaturation at 95°C for 2 minutes, followed by 45 cycles of denaturation at 95°C for 15 seconds, annealing at 58°C for 30 seconds, and extension at 72°C for 30 seconds with a cut-off cycle threshold of 36 Ct.

The detection of IgM antibodies against *O. tsutsugamushi* in serum was performed using a commercial ELISA kit (InBios International Inc., Seattle, WA, USA) for the detection of scrub typhus IgM as per the manufacturer’s instructions. An Optical density (OD) of >0.5 for IgM by ELISA was considered positive.

Sequencing for *Orientia tsutsugamushi*

Using a high-fidelity Taq polymerase (Thermo Fisher Scientific), a nested PCR was performed for the 56 kDa TSA gene region in samples that tested positive for scrub typhus DNA in real-time PCR. Three of the four main variable areas were covered by the gene sequence that was thus acquired. Ruang-areerate et al. describe the use of two sets of primers for sequencing. F (5′-AGC GCTAGG TTT ATT AGC AT) and RTS8 (5′-AGG ATT AGA GTG TGG TCCTT) were the inner primers, and JG-OtF584 (5′-CAA TGT CTG CGT TGT CGT TGC) and RTS9 (5′-ACAGAT GCA CTA TTA GGC AA) were the outside primers [9]. Using the BigDye Terminator Cycle Sequencing Kit (Applied Biosystems, Foster City, CA, USA), the PCR product was sequenced unidirectionally.

The sequences from this work have been assigned GenBank accession numbers that range from PP393459 to PP393473.

Statistical analysis

Data were entered into an MS Excel spreadsheet (Microsoft Corp., Redmond, WA, USA) and analyzed using IBM SPSS Statistics for Windows, version 20.0 (IBM Corp., Armonk, NY, USA). For the descriptive data, the mean, standard deviation, and percentage were calculated. A chi-square test was applied for comparison of categorical variables. 

## Results

In this study, 264 consecutive pediatric patients with AES were enrolled between August 2018 and November 2019. The mean age of presentation was 6.86 ± 3.44 years (0.3-14), with a male-to-female sex ratio of 1.11:1. 

**Figure 1:**
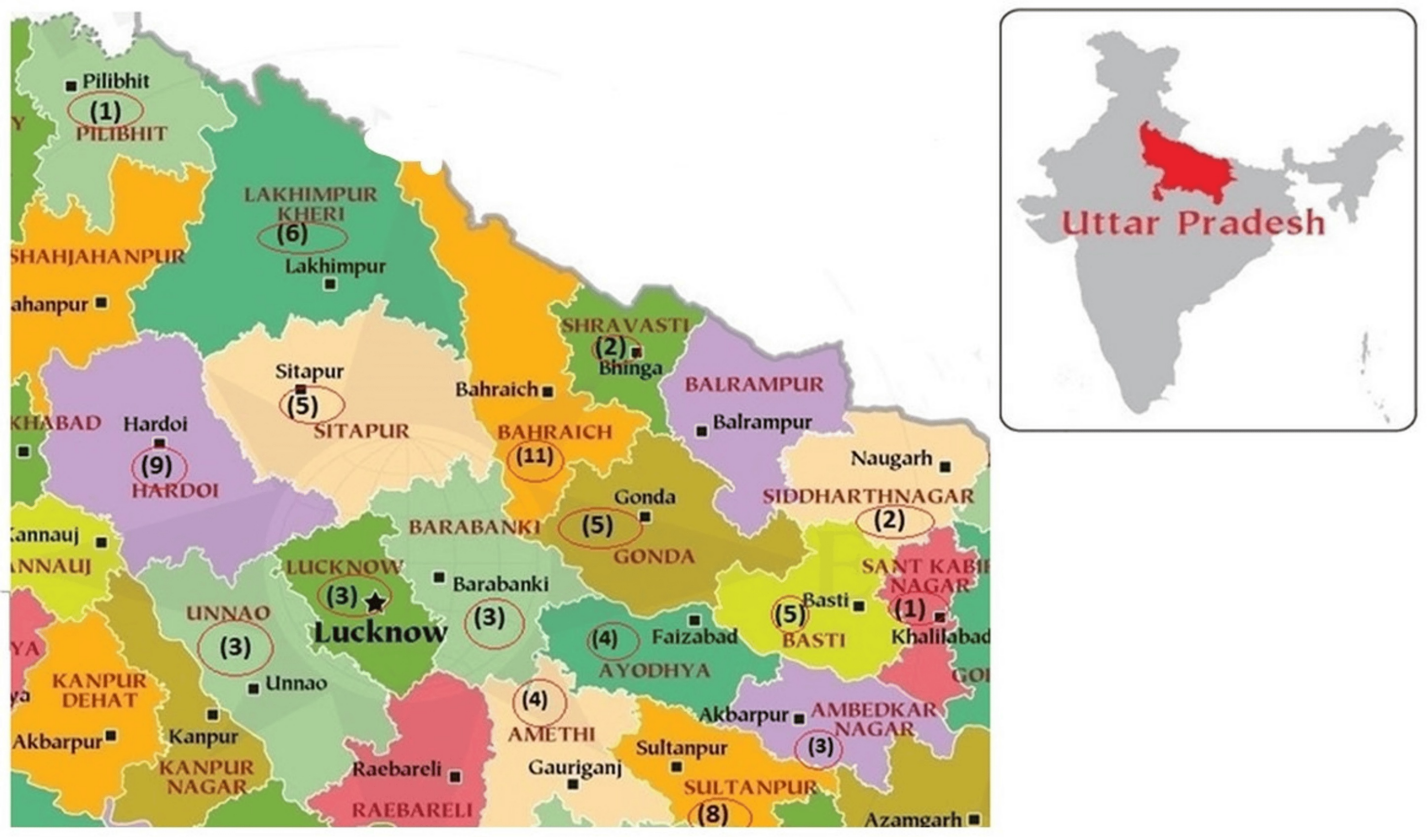
District-wise distribution of acute encephalitis syndrome cases positive for scrub typhus

A total of 78 (29.5%) samples were positive through a combination of PCR and ELISA using different sample types, including CSF, serum, and PBMC. CSF-PCR was positive in 11 cases (4.17%), PBMC-PCR identified 25 (9.47%), and ELISA IgM identified 70 (26.52%) cases. ELISA positivity was significantly higher than PCR in CSF and PBMC. Two cases (0.76%) were positive for both CSF-PCR and ELISA IgM, two (0.76%) were positive for both PBMC-PCR and CSF-PCR, and 15 cases (5.68%) were positive for PBMC-PCR and ELISA IgM. Additionally, four cases (1.52%) were positive for all three tests (PBMC-PCR, CSF-PCR, and ELISA IgM). A significant proportion of cases (n = 186, 70.45%) were negative for all tests (Table 1). The district-wise distribution of these 78 scrub-positive cases is shown in Figure 1.

**Table 1:** Positivity of different tests/combination of tests among children presenting with acute encephalitis syndrome PCR, polymerase chain reaction; ELISA, enzyme-linked immunosorbent assay; CSF, cerebrospinal fluid; PBMCs, peripheral blood mononuclear cells; IgM, immunoglobulin M; N/A, not applicable

Test/sample combination of test	Positive cases	Percentage	p-value
CSF-PCR	11	4.17%	<0.001
PBMC-PCR	25	9.47%	
ELISA IgM	70	26.52%	
CSF-PCR + ELISA IgM	2	0.76%	N/A
PBMC-PCR + CSF-PCR	2	0.76%	N/A
PBMC-PCR + ELISA IgM	15	5.68%	N/A
PBMC-PCR + CSF-PCR + ELISA IgM	4	1.52%	N/A
Total positive cases	78	29.50%	N/A
Total AES cases	264	-	N/A

**Figure 2:**
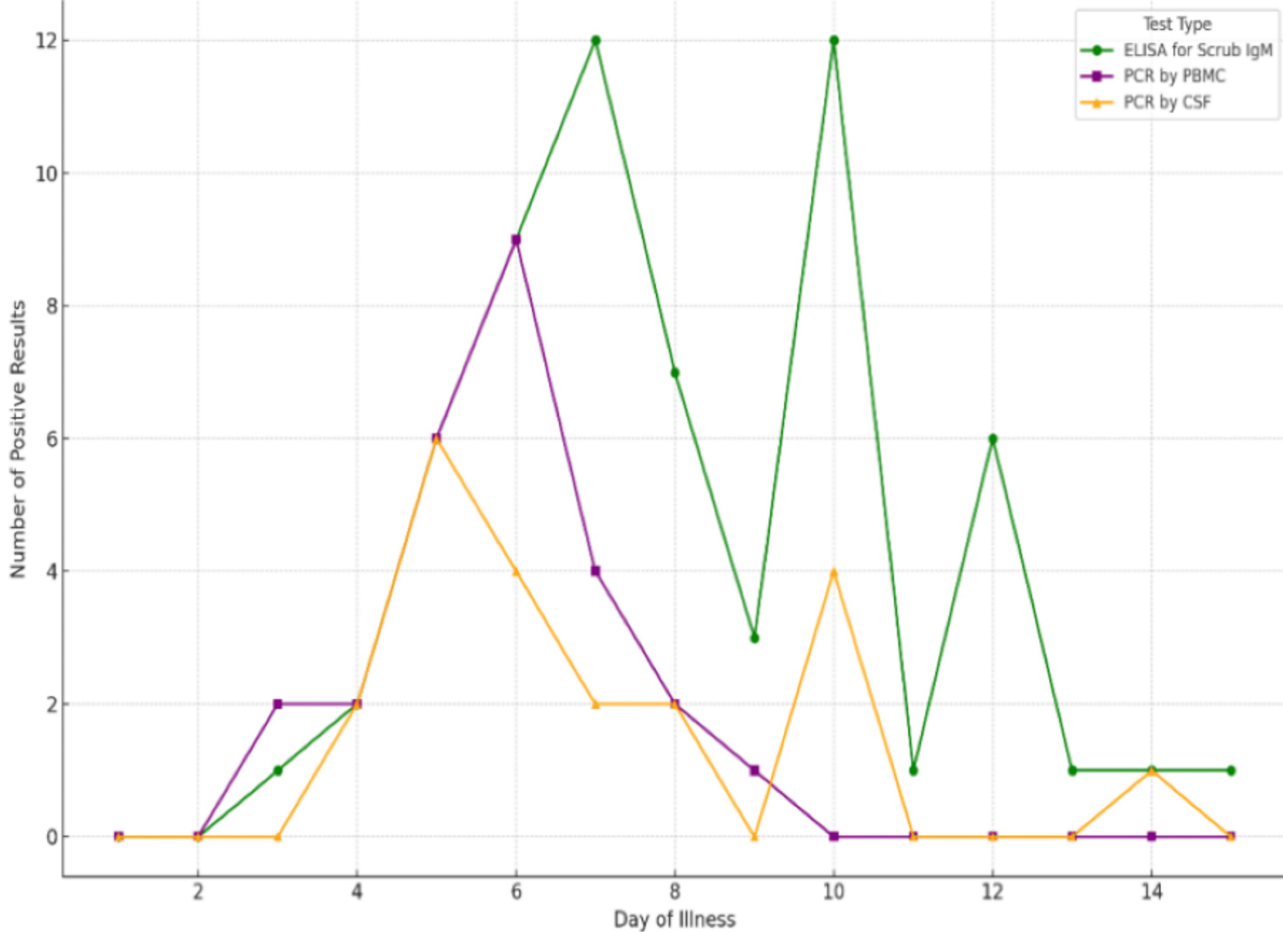
Line graph illustrating the distribution of positive results across different days of illness for three diagnostic tests

ELISA for scrub typhus IgM, PCR by PBMC, and PCR by CSF displayed distinct trends in detecting scrub typhus based on the day of illness. The ELISA for scrub typhus IgM test showed a notable peak in positive results on the sixth day of illness, with the number of positive cases reaching 12. A secondary peak was observed around the 10th day, though the number of positive results declined sharply afterward, with very few cases detected by the 14th day (Figure 2).

In contrast, the PCR by PBMC test peaked earlier, around the fifth day of illness, detecting approximately nine positive cases. After this peak, the number of positive results from PBMC testing dropped significantly and remained low for the rest of the illness duration.

The PCR by CSF test detected fewer positives overall, with a small peak on the sixth day, identifying about three positive cases, followed by a slight increase around the ninth day. However, the number of positive cases detected by CSF remained consistently low throughout the illness period.

**Figure 3:**
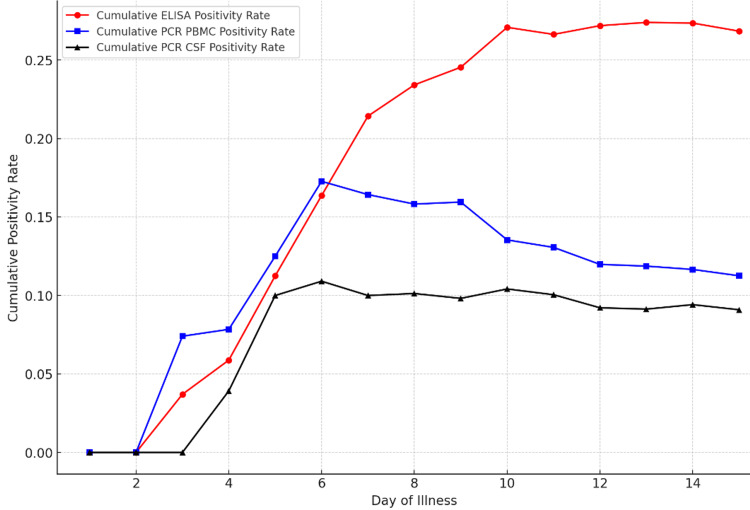
Cumulative positivity rates for three diagnostic tests according to day of illness

The ELISA for the scrub typhus IgM test demonstrates the highest cumulative positivity, with a sharp increase starting around the fourth day of illness. It reaches its peak between the ninth and 10th days, stabilizing at approximately 0.25, making it the most sensitive test over time. Similarly, the PCR by PBMC test shows a rapid rise in positivity beginning between the third and fourth days, but it plateaus by the sixth day, achieving a cumulative positivity rate close to 0.15 by the eighth day. On the other hand, the PCR by CSF test displays the lowest cumulative positivity rate, with a more gradual increase that peaks around 0.10 by the seventh or eighth day and then remains steady. This comparison highlights the varying effectiveness of each test, with ELISA for scrub typhus IgM emerging as the most responsive across the duration of the illness, followed by PCR by PBMC and PCR by CSF (Figure 3).

Among scrub-positive patients, a total of 25 tested positive for scrub typhus using real-time PCR with PBMC samples (Table 1). Of these 25 patients, 15 with favorable cycle threshold (Ct) values (26-32) were selected for further analysis using nested PCR to amplify the 56 kDa antigen of *O. tsutsugamushi*. Additionally, we intended to amplify the 56 kDa gene for a higher Ct value, that is, more than 33 Ct, but we were unable to do so.

**Figure 4:**
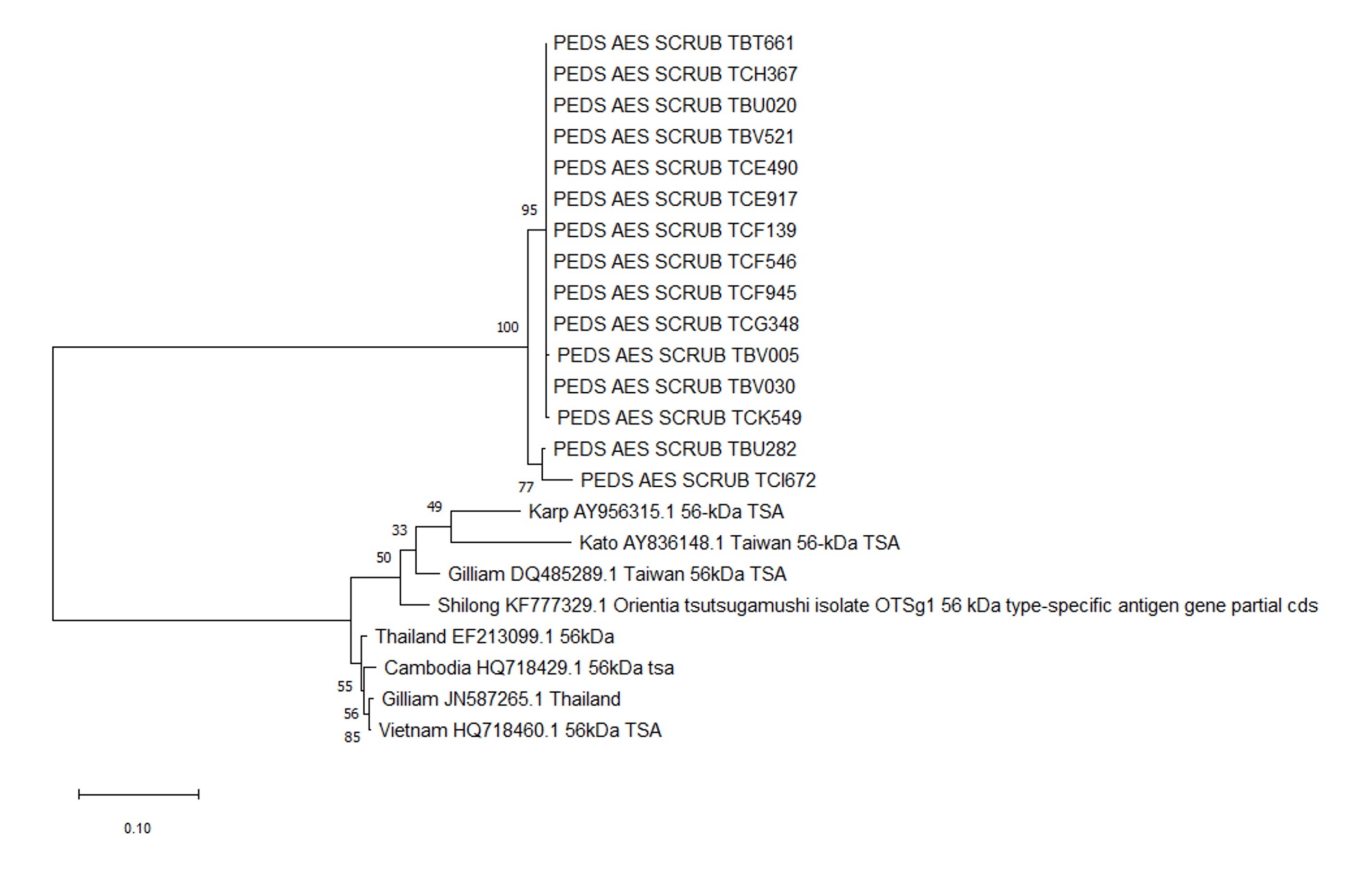
Phylogenetic tree based on the 56 kDa TSA gene ORF

Phylogenetic analysis revealed that most isolates were closely related to the Gilliam genotype from Vietnam and Thailand. The resulting 15 amplicons were sequenced and phylogenetically compared to prototype strains of *O. tsutsugamushi*, as well as strains reported from India and neighboring countries (Figure 4).

Fifteen amplicons with good Ct value from 26 positive samples with PBMCs were subjected to phylogenetic analysis, and the results were compared to prototype strains of *O. tsutsugamushi*, strains from India, and strains Keto, Karp, and Gilliam from nearby nations (retrieved from NCBI for phylogenetic tree construction).

Evolutionary history was inferred by using the maximum likelihood method, and Tamura-Nei model evolutionary analyses were conducted using the software Molecular Evolutionary Genetics Analysis (MEGA) 11.

## Discussion

This study integrates both molecular (PCR) and serological (ELISA) diagnostic techniques to assess scrub typhus in pediatric AES cases, ensuring a more comprehensive understanding of disease detection across different biological samples. Standard protocols were followed for all laboratory tests. By analyzing CSF, serum, and PBMC samples, the study effectively highlights the variations in diagnostic sensitivity at different stages of illness, demonstrating the early detection capability of PCR in PBMC and the higher cumulative positivity of ELISA in serum samples. The large sample size (264 pediatric patients) enhances the reliability of the findings, while the inclusion of phylogenetic analysis offers valuable insights into the genetic diversity of *O. tsutsugamushi* strains circulating in the region. Furthermore, the study’s strategic comparison of PCR and ELISA underscores the importance of a combined diagnostic approach, optimizing early detection and treatment outcomes. These findings have significant clinical implications for refining diagnostic protocols, improving patient management, and guiding future research on scrub typhus in endemic regions.

The findings of this study underscore the varying efficacy of diagnostic methods for detecting scrub typhus, particularly in pediatric patients with AES. ELISA for scrub typhus IgM emerged as the most sensitive diagnostic tool, with its cumulative positivity rate significantly increasing from the fourth day of illness and stabilizing between the ninth and 10th days. This result is consistent with other studies that have identified ELISA as a reliable method for detecting IgM antibodies during the acute phase of scrub typhus [10,11]. Blacksell et al. demonstrated that IgM ELISA exhibits high sensitivity (93.5%) and specificity (91.1%) in diagnosing acute scrub typhus, making it a reliable method for detecting antibodies in endemic regions [12]. Koraluru et al. also found that IgM ELISA had a sensitivity of 92% and specificity of 95%, further reinforcing its effectiveness in early disease detection [13]. Also, in a meta-analysis, it was highlighted that IgM ELISA remains a highly sensitive and specific diagnostic tool for scrub typhus across multiple endemic regions, supporting its role as a primary test in clinical settings [14].

On the other hand, PCR by PBMC showed high effectiveness in the early stages of illness, particularly between the third and fourth days. This rapid detection capability aligns with the molecular principles of PCR, which can detect *O. tsutsugamushi* DNA before the body begins producing antibodies [15]. Previous studies have emphasized the importance of early PCR testing for improving patient outcomes, especially in severe cases where early intervention is critical [16,17].

However, the lower cumulative positivity rate observed with PCR by CSF suggests that this method may be less reliable for diagnosing scrub typhus in AES cases. The lower bacterial load in CSF compared to blood samples could explain this, as seen in other research where CSF samples yielded less consistent results [18,19]. While PCR by CSF can serve as a useful confirmatory test, it should not be relied upon as the sole diagnostic method.

The overall positivity rate for scrub typhus in AES cases in this study was 29.5%, consistent with findings from other studies conducted in endemic regions, where scrub typhus is increasingly recognized as a major cause of AES [20,21]. This highlights the need for greater awareness and enhanced diagnostic capabilities, particularly in pediatric populations, as delayed diagnosis and treatment of scrub typhus are associated with significant pediatric mortality rates ranging from 7% to 15% [22,23].

These results emphasize the importance of a strategic approach to diagnostic testing, taking into account the stage of illness and the type of sample available. ELISA for scrub typhus IgM is best suited as a primary diagnostic tool for patients presenting later in the illness, while PCR by PBMC is more appropriate for early detection. These findings align with global recommendations advocating a combination of serological and molecular diagnostics to ensure accurate and timely diagnosis [2,24].

The phylogenetic analysis of scrub typhus revealed many Gilliam-like strains, which were found to be comparable to those isolated from Cambodia, Vietnam, and Thailand, as determined through sequencing analysis. In 2015, research findings indicated that there were comparable proportions of Karp-like and Kato-like strains in northern India [24]. However, our study did not find significant similarity between the Karp strain and the Kato strain, potentially due to the limited number of samples available. Gilliam-like strains have been found in Vellore and Shillong, but only a few have reported this from northern India. Bakshi et al. documented the presence of Gilliam-like strains in northern India in the year 2007 [25]. Studies of small animals and mites from central India have also found that Karp and Gilliam strains predominate [26].

To incorporate *O. tsutsugamushi* strains into the creation and assessment of diagnostic assays and immunizations, it is imperative to know the strains that are currently in circulation in each region. Vaccine trial failures have resulted from vaccines’ inability to protect against multiple prevalent strains [27,28]. Further analysis of this pathogen biology is not feasible due to a lack of whole genome sequence data from India and insufficient genomic resources.

Given the limitations of each method, further research is recommended to explore advanced diagnostic techniques, such as next-generation sequencing (NGS) or multiplex PCR assays, which may offer higher sensitivity and specificity across different sample types and stages of illness [29]. Moreover, increased clinician awareness regarding the optimal timing and selection of diagnostic tests is crucial for improving patient outcomes in scrub typhus-endemic regions.

The study’s single-center focus and limited sample size may limit the generalizability of the findings. Differences in patient illness stages at presentation may have influenced test positivity rates. The sensitivity and specificity of different tests could not be calculated due to the lack of a gold standard test in the present study. Additionally, the study did not include eschar samples, which could provide further diagnostic insights.

## Conclusions

Study findings indicate that ELISA for scrub typhus IgM has a high positivity rate in comparison to PCR for the detection of scrub typhus in children with AES, particularly for detecting scrub typhus during the later stages of illness, while PCR, especially using PBMC samples, is valuable for early detection. The overall positivity rate of 29.5% underscores the significant impact of scrub typhus in AES cases in endemic regions. Improved diagnostic strategies, including the integration of multiple sample types and advanced molecular techniques, are essential to enhance early detection and treatment outcomes for this vulnerable pediatric population.
